# Different pancreatic cancer microenvironments convert iPSCs into cancer stem cells exhibiting distinct plasticity with altered gene expression of metabolic pathways

**DOI:** 10.1186/s13046-021-02167-3

**Published:** 2022-01-21

**Authors:** Ghmkin Hassan, Toshiaki Ohara, Said M. Afify, Kazuki Kumon, Maram H. Zahra, Xiaoying Fu, Mohamad Al Kadi, Akimasa Seno, David S. Salomon, Masaharu Seno

**Affiliations:** 1 Department of Biotechnology and Drug Discovery, Graduate School of Interdisciplinary Science and Engineering in Health Systems, Okayama University, 3.1.1 Tsushima-Naka, Kita, Okayama, 700-8530 Japan; 2 Department of Genomic Oncology and Oral Medicine, Graduate School of Biomedical and Health Science, Hiroshima University, Hiroshima, 734-8553 Japan; 3 Department of Pathology and Experimental Medicine, Medical School, Okayama University, Okayama, 700-8558 Japan; 4 Division of Biochemistry, Chemistry Department, Faculty of Science, Menoufia University, Shebin El Koum-Menoufia, 32511 Egypt; 5 Department of Pathology, Tianjin University of Traditional Chinese Medicine, Tianjin, 300193 China; 6 Department of Bacterial Infections, Research Institute for Microbial Diseases, Osaka University, Osaka, 565-0871 Japan; 7 The Laboratory of Natural Food and Medicine, Co., Ltd., Okayama, 700-8530 Japan; 8 Mouse genetics program, Center for Cancer Research, National Cancer Institute, Frederick, MD 21702-1201 USA

**Keywords:** Cancer stem cells, iPSCs, Conversion, Plasticity, Tumorigenesis, Pancreatic cancer microenvironments

## Abstract

**Background:**

Cancer stem cells (CSCs) are generated under irregular microenvironment in vivo, of which mimic is quite difficult due to the lack of enough information of the factors responsible for cancer initiation. Here, we demonstrated that mouse induced pluripotent cells (miPSCs) reprogrammed from normal embryonic fibroblasts were susceptible to the microenvironment affected by cancer cells to convert into CSCs in vivo.

**Methods:**

Three different pancreatic cancer line cells, BxPC3, PANC1, and PK8 cells were mixed with miPSCs and subcutaneously injected into immunodeficient mice. Tumors were evaluated by histological analysis and cells derived from iPSCs were isolated and selected from tumors. The isolated cells were characterized for cancer stem cell characters in vitro and in vivo as well as their responses to anticancer drugs. The impact of co-injection of iPSCs with cancer cells on transcriptome and signaling pathways of iPSCs was investigated.

**Results:**

The injection of miPSCs mixed with human pancreatic cancer cells into immunodeficient mice maintained the stemness of miPSCs and changed their phenotype. The miPSCs acquired CSC characteristics of tumorigenicity and self-renewal. The drug responses and the metastatic ability of CSCs converted from miPSCs varied depending on the microenvironment of cancer cells. Interestingly, transcriptome profiles of these cells indicated that the pathways related with aggressiveness and energy production were upregulated from the levels of miPSCs.

**Conclusions:**

Our result suggests that cancer-inducing microenvironment in vivo could rewire the cell signaling and metabolic pathways to convert normal stem cells into CSCs.

**Supplementary Information:**

The online version contains supplementary material available at 10.1186/s13046-021-02167-3.

## Background

Cancer initiation and progression are a multi-step process and have been linked to the inflammatory microenvironment which contains secreted cytokines, pro-inflammatory factors, and extracellular matrix components [1]. Chronic inflammation followed by tissue repair involves different types of cells such as immune cells and fibroblasts. Upon various stimuli, these cells produce factors such as proteases, growth factors, and chemokines which play essential roles in the growth and survival of cancer cells [2, 3]. The chronic inflammatory microenvironment, disrupting tissue homeostasis for a long period, creates a tumor inducing niche. Then, the cells in the microenvironment will adapt to the shifted homeostasis changing the phenotypes close to cancer cells. Once a cell associated with cancer arises, the cell, in turn, becomes the source of the microenvironment that evolves cancer mass [4]. Since the cellular and biochemical processes involved in wound healing share a good similarity with those in the growth and development of tumors, a tumor is often described as “a wound that does not heal” [5].

Stem or progenitor cells residing in almost all tissues are defined by their characters of self-renewal and differentiation capacity. The transformation of normal cells into neoplastic cells was traditionally suggested as an accountable event for tumorigenesis where normal stem cells acquire malignancies and become cancer stem cells (CSCs) [6]. CSCs are defined as cancer cells with the self-renewal and differentiation abilities as same as normal stem cells. In this context, CSC generation can be explained by the chronic inflammatory microenvironment resulted from tissue repair processes that are repeated in the damaged tissues [7]. The concept of stemness has been extended to theorize the capacity of differentiation coupled with the self-renewal of CSCs as a source of the heterogeneity present in tumors [8]. In decades of research, CSCs were shown to have the most aggressive phenotype among cancer cell populations as well as their responsibilities of drug resistance and cancer relapse. CSCs originally defined as very small populations of cells, less than 1%. CSCs were identified in different cancer types such as blood malignancy, breast, liver, lung, brain, pancreatic cancers, and so on [8, 9] . Currently, CSCs are being isolated from tumor samples directly by applying affinity-based methods such as flow cytometry or magnetic-activated cell sorting. CSCs can also be enriched from cancer cell lines by affinity-based or 3D culture methods. So far, CSC isolation is a relatively difficult task due to their low rate and difficulties in stable maintenance in cell culture, and this has been obstructing large-scale research and analysis. Moreover, not all CSC markers, which may vary depending on tumor tissue and the heterogeneity in CSC populations, have been identified [10–12].

Induced pluripotent stem cells (iPSCs) technology have been raised as a potential tool to obtain pluripotent stem cells from adult individuals. The iPSCs facilitate modeling personalized models for different types of diseases including cancer [13, 14]. The advantage of iPSC technology is the potential to provide cancer or CSC models for individuals who have risks of cancer even before cancer develops [14, 15]. Several groups have generated cancer models from iPSCs by genetic manipulation. While these CSC developed by genetic manipulation will apparently provide novel tools for oncology research, the epigenetic effects inducing cancer are predominantly neglected in these models and do not give insights into CSC induction [13–17]. Previously, we presented a method to obtain CSCs converted from iPSCs by culturing iPSCs in the presence of conditioned media (CM) from cancer cells for almost one month. The iPSCs were successfully converted into CSC models using CM from breast, liver, pancreatic, and lung cancer cell line cells by that approach [18–20]. The CM, therefore, was proposed as a mimicker of the cancer induction microenvironment. The major issues for that method are the variability of CM depending on cancer cells, the long culture time of iPSCs in CM, and the absence of cell-cell interactions with different types of cells such as immune cells and fibroblasts. Therefore, this method still needs to be optimized. Taking these into consideration, in this study, we attempted to induce CSCs from miPSCs in vivo. Here, we report that the pancreatic cancer microenvironments presented by pancreatic cancer cells could convert normal stem cells, iPSCs, into CSCs, by injection mixtures of cancer cells and miPSCs into immunodeficient mice. CSCs converted from iPSCs were characterized by their self-renewal, differentiation, and high tumorigenicity abilities. Moreover, the obtained CSCs exhibited variant plasticity depending on different cancer microenvironments and the changes in gene expression associated with different metabolic pathways were found.

## Material and methods

### Cell culture

The miPSCs (iPS-MEF-Ng-20D-17, Lot No. 012, Riken Cell Bank, Tokyo, Japan) which were designed to express the green fluorescent protein (GFP) and puromycin (puro) resistant genes under the control of the Nanog promoter, were maintained on mitomycin-C-treated mouse embryonic fibroblasts (Reprocell, Japan) and then passaged to feeder-free condition according to Chen et al. [20] The miPSCs maintained in the miPS medium consisting of DMEM, 15% fetal bovine serum (FBS) (Thermo Fisher Scientific, MA), 0.1 mM non-essential amino acid (NEAA), (Thermo Fisher Scientific, MA), 2 mM L-glutamine (Nacalai Tesque, Japan) and 0.1 mM 2-mercaptoethanol (β-ME) (Sigma-Aldrich, MO) supplemented with 100 U/ml penicillin and 100 μg/ml streptomycin (Wako, Japan). The cells were maintained in the presence of 1000 U/mL leukemia inhibitory factor (LIF) (Merck Millipore, MA).

The human pancreatic cancer cell lines BxPC-3 (ATCC, VA), PANC-1, and PK-8 (RIKEN, Japan) cells were cultured in RPMI-1640 (Wako, Japan) containing 10% FBS supplemented with 100 U/ml penicillin and 100 μg/ml streptomycin. The Balb/c 3 T3 (ATCC, VA) were cultured in DMEM containing 10% FBS with 100 U/ml penicillin and 100 μg/ml streptomycin.

For the primary culture of cells from tumors, tumor tissues were washed with phosphate-buffered saline (PBS), finely minced with a razor blade in Hank’s balanced salt solution (HBSS) and then 1 ml of tissue suspension was transferred into 15-ml tubes containing 1 ml of dissociation buffer consisting of 0.1% collagenase, 0.25% trypsin, 20% KnockOut Serum Replacement (Thermo Fischer Scientific, MA), and 1 mM of CaCl_2_ (Sigma-Aldrich, MO) in PBS. The cell suspensions were then incubated at 37 °C for 1 h with shaking. After digestion, 3 mL of DMEM with 10% FBS was added to stop digestion. The suspension was then centrifuged at 400×g for 5 min., the supernatants were discarded, cell pellets were suspended in 2 ml of miPS medium and then the number of cells was counted. The cells were then seeded into gelatin-coated dishes at a density of 5 × 10^5^ cells/dish in miPS medium without LIF. For the selection of green fluorescent protein (GFP) positive cells, cells were treated with 1.5 μg/ml puromycin for 1 week.

To test the sensitivity of BxPC-3, PANC-1, and PK-8 to puromycin, 5 × 10^4^ cells were seeded in 12-well plates and when cells reached 70–80% confluence, cells were treated with 1.5 μg/ml puromycin for 4 days. After the media were discarded, cells were washed with PBS. Then fresh media without puromycin were added to wells and the cells were kept in a 37 °C incubator with 5% CO_2_ for 2 weeks with medium change twice a week. Cell morphology and GFP fluorescence were observed and photographed under an Olympus IX81 microscope equipped with a light fluorescence device (Olympus, Japan).

### Animal experiments

The 4-week-old NOD-SCID mice were purchased from Charles River, Japan and kept under pathogen-free conditions. Mice were divided into seven groups, with three mice in each group. Three groups were used for injection of BxPC-3, PANC-1, and PK-8 cells at a dose of 5 × 10^6^ cells/mice. One group was used for injection of 0.5 × 10^6^ of miPSCs. Three groups were injected with cell mixtures consisting of 5 × 10^6^/0.5 × 10^6^ of PANC1 cells and miPSCs (miPS-Panc1), 5 × 10^6^/0.5 × 10^6^ of BxPC3 cells and miPSCs (miPS-Bxpc3) or 5 × 10^6^/0.5 × 10^6^ of PK8 cells and miPSCs (miPS-Pk8). Cells were suspended into 100 μl of PBS with 20% Matrigel matrix (Corning, NY) and subcutaneously injected into mice. After 6 weeks of injection, mice were euthanized with 5% of isoflurane through inhalation and the cervical was dislocated to ensure the death of mouse and tumors were excised.

Further injection of converted cells from miPSCs was performed in the pancreases of 4-week-old Balb/c nu/nu female mice (Charles River, Japan). 5 × 10^5^, 5 × 10^4^, or 5 × 10^3^ of GFP positive cells isolated from the tumors of miPSCs-Bxpc3, miPSCs-Panc1, and miPSCs-Pk8 and selected with puromycin, designated as miBx, miPa, and miPK cells, respectively, were suspended into 40 μl of PBS with 10 μl Matrigel matrix. Mice were anesthetized for surgery with isoflurane, 3% for induction and 1.5% for maintenance, and 5 × 10^5^, 5 × 10^4^ or 5 × 10^3^ cells were injected into the pancreases. Four weeks after the injection of 5 × 10^5^ cells, and 5 weeks after the injection of 5 × 10^4^ or 5 × 10^3^ cells, mice were sacrificed. Then, the tumors were excised and organs were examined for metastases.

Groups of 4-week-old Balb/c nu/nu female mice were subcutaneously injected with 1 × 10^5^ of mi-BxCS, mi-PaCS, and mi-PKCS cells, which prepared from primary cultures of pancreases tumors. Cells were suspended into 100 μl of PBS with 20% Matrigel matrix. Injected mice were monitored for 5 weeks, and then sacrificed. Mice injected with miPSCs were used as controls and each group contained three mice. The animal experiments were conducted following Okayama University guidelines after the approval by the ethics committee for animal experiments at Okayama University, approval IDs OKU-2019496, OKU-2019497, and OKU-2020625.

### Histopathology

Tumors tissues were fixed in formalin (Wako, Japan), embedded in paraffin (Wako, Japan), and sectioned with the depth of 5 μm by a RM2255 microtome (Leica, Germany). For the nuclear/cytoplasm staining, the sections were stained with hematoxylin and eosin Y (Sigma-Aldrich, MO). For immunohistochemistry, slides of tumor sections were immersed in sodium citrate buffer consisting of 10 mM sodium citrate pH 6.4 and 0.05% Tween 20 and autoclaved to retrieve antigens. The endogenous peroxidase activity was blocked with 0.3% hydrogen peroxide and then incubated for 1 h with normal serum for blocking. The slides were incubated at 4 °C overnight with primary antibodies (Supplementary Table 7). After washing with PBS, slides were immunostained with VECTASTAIN® Elite® ABC kit (Vector Laboratory, Germany) according to the instruction provided by the manufacturer. The signals were visualized by 3.3′-Diaminobenzidine (DAB) a peroxidase substrate (Vector Laboratory, Germany) and slides were counterstained with Mayer’s Hematoxylin (Sigma-Aldrich, MO) and mounted with Softmount (Wako, Japan). Slides were observed and photographed by an FSX100 microscope (Olympus, Japan) and the immunoreactive areas were quantified with ImageJ software (NIH, MD).

### PCR and reverse transcription-quantitative PCR (RT-qPCR)

The genomic DNA was isolated from cells using Blood & Cell Culture DNA Mini Kit (Qiagen, Germany) according to the manufacturer instructions. The PCR reactions were performed with 200 ng of DNA, Taq 2X Master Mix (New England Biolabs, MA), and a primer set specific for the Alu sequence (Supplementary Table 8). The PCR products were applied on 1.5% agarose gel electrophoresis and visualized with ethidium bromide (Sigma-Aldrich, MO). The 100 pb ladder (Takara Bio, Japan) was used as a size marker.

Total RNA was isolated with TRIzol (Thermo Fisher Scientific, MA), treated with DNase I (Promega, WI), and A260/A280 and A260/A230 ratios were measured with NanoDrop to assess RNA purity. Two μg of total RNA was reverse transcribed with GoScript™ Reverse Transcription System (Promega, WI) according to the manufacturer instructions and RT-qPCR reactions were conducted by Light Cycler 480 II using Light Cycler 480 SYBR Green I Master Mix (Roche Diagnostics, Germany). Gene expression levels were normalized to that of the beta actin (Actb) gene. The primer sets used for the PCR and RT-qPCR reactions are listed in (Supplementary Table 8).

### Flow cytometry

To assess the percentage of GFP positive cells, cells were trypsinized, washed with cold PBS, counted, and 1 million cells were suspended in PBS containing 10% FBS and applied to Accuri C6 Plus flow cytometer (BD Bioscience, CA). One million cells were also blocked with mouse FcR blocking reagent (Miltenyi Biotec, Germany) and then incubated with fluorescence-conjugated primary antibodies (Supplementary Table 7). After incubation, cells were washed, suspended in PBS, and applied to Accuri C6 Plus flow cytometer. The data were analyzed by Flowjo software (FlowJo, LLC, ORE) by gating live and single cells excluding the aggregates and dead cells.

### Sphere-formation and extreme limiting dilution assays

To assess cell’s ability to form spheroids, 5 × 10^4^ cells were seeded into ultra-low attachment 35-mm culture dishes (Prime Surface Dish, Sumitomo Bakelite, Japan) with serum-free media consisting of DMEM, 0.1 mM β-ME, 2 mM L-glutamine, 0.1 mM NEAA, and ITS-X (Wako, Japan) supplemented with penicillin/streptomycin. Cells were then incubated at 37 °C in 5% CO_2_ for 7 days and formed spheres were photographed using an Olympus IX81 microscope equipped with a light fluorescence device (Olympus, Japan).

For extreme limiting dilution assay (ELDA), 500, 100, 50, and 10 cells/well were seeded in either 24-well plates for 500 cells or 96-well ultra-low attachment v-bottom plates (Sumitomo Bakelite, Japan) for 100 or fewer numbers of cells in the same serum-free media as above for 7 days. The number of wells containing spheroids was counted and the frequency of spheroid forming cells was calculated with ELDA software available at http://bioinf.wehi.edu.au/software/elda/index.html.

### Tube-formation assay

The 7.5 × 10^4^ cells were resuspended in EBM2 media, an endothelial basal medium (Lonza, Switzerland) supplemented with 1 μg/ml ascorbic acid, 0.2 μg/mL hydrocortisone, 22.5 μg/mL heparin, and 2% FBS (Lonza, Switzerland). Cells were seeded in 24-well plates coated with Matrigel matrix (Corning, NY) and incubated at 37 °C in 5% CO_2_. After 24 h, images of tube structures were taken by an Olympus IX81 microscope (Olympus, Japan) and images were analyzed with the angiogenesis analyzer tool in Image J.

### Immunofluorescence

Cells were seeded on round coverslips inserted in 24-well plates coated with Matrigel matrix as same as mentioned in the section of tube formation. After 24 h, cells were fixed with 4% paraformaldehyde (Wako, Japan) for 20 min at 25 °C, and washed with PBS. Cells were then blocked with PBS supplemented with 10% FBS for 1 h and then incubated with the primary antibody at 4 °C overnight (Supplementary Table 7). At the end of incubation, cells were washed with PBS and incubated with the secondary antibody (Supplementary Table 7). for 1 h at 25 °C. The coverslips were then removed and mounted on glass slides using VECTASHIELD® antifade mounting medium with DAPI (Vector Labs, Germany). Images were taken by the FSX100 microscope (Olympus, Japan).

### Invasion assay

Cell invasion capability was assessed using 24-well Corning BioCoat™ Matrigel Invasion Chamber (Corning, NY). The 5 × 10^4^ cells were seeded into the insert according to the instructions of the manufacturer. After 48 h of incubation at 37 °C and 5% CO_2_, the media were discarded, and inserts were washed with PBS and stained with Diff-Quik kit (Sysmex, Japan) according to the instructions provided with BioCoat™ Matrigel Invasion Chamber. The invaded cells were counted and photographed by the FSX100 microscope (Olympus, Japan).

### Scratch assay

Cells were trypsinized, counted, and 10^6^ cells were seeded on a gelatin-coated 60-mm culture dish in miPS medium. Cells were incubated until they formed monolayers. The scratches were performed using 200-μL pipette tips, cells were washed with PBS, and DMEM with 0.5% FBS media was added to cells. The scratched areas were monitored and photographed at the time points of 0, 12, and 24 h after scratching. The areas of healing relative to the beginning were calculated with ImageJ software.

### MTT assay

The cells were seeded in 96-well plates (5000 cells/well) with miPS media with different concentrations of gemcitabine hydrochloride (Tokyo Chemical Industry, Japan) or doxorubicin (Wako, Japan) and plates were incubated at 37 °C and 5% CO_2_. After 72 h, cell viability was evaluated by thiazolyl blue tetrazolium blue (MTT, Sigma-Aldrich, MO). MTT solution was added at 0.5 mg/mL concentration into wells and incubated for 4 h at 37 °C in 5% CO_2_. The formazan crystals formed after incubation were dissolved with DMSO (Sigma-Aldrich, MO) and the absorbance was measured at 570 nm by an MTP-800 Lab microplate reader (Corona Electric, Japan). The IC_50_ values were estimated from the survival curves.

### Differentiation of CSCs

In order to allow 3D structure formation consisting of both undifferentiated and differentiated cells, 2 × 10^6^ cells were seeded in non-coated Petri dishes (AS ONE, Japan) as the semi-adherent condition for 3D culture. The cells were cultured in serum-free media consist of DMEM, 0.1 mM β-ME, 2 mM L-glutamine, 0.1 mM NEAA, penicillin/streptomycin, ITS-X supplement, 20 ng/ml epidermal growth factor (EGF), and 10 ng/ml fibroblast growth factor 2 (FGF2) (Katayama chemical, Japan). Cells were incubated at 37 °C in 5% CO_2_ for 7 days and 3D structures were photographed by an Olympus IX81 microscope equipped with a light fluorescence device (Olympus, Japan). To culture 3D structures on coated dishes, the media were collected from Petri dishes, centrifuged at 400×g for 5 min, pellets were washed with PBS and suspended in 1 ml of miPS medium. The 200 μl of pellets were cultured in 60 mm dishes coated with gelatin in miPS medium. Two days after seeding, images of cells were captured by an Olympus IX81 microscope equipped with a light fluorescence device.

### RNA sequencing and bioinformatic analysis

Total RNA was isolated from cells and treated with DNase I as mentioned above. The RNA integrity was tested using Agilent Bioanalyzer 2100 and samples with RNA Integrity Number (RIN) more than 7 were chosen. Sequencing libraries were prepared with NEBNext Ultra II RNA Library Prep Kit (New England Biolabs, MA). The sequencing of the 150-bp paired-end was performed with Novaseq6000 (Illumina, CA). The bioinformatic analysis was performed with Galaxy <usegalaxy.org>. The Tophat tool was used to align reads with the mouse reference genome mm9, the Cufflinks was used to assembles transcripts and assess their abundances. Then, transcripts were merged with Cuffmerge, quantitated by Cuffquant, and normalized by Cuffnrorm. The Cuffdiff was used for differential expression analysis between samples. The differential expressed genes (DEGs) were chosen based on cutoff fold change ≥2 and false positive rate (FDR) < 0.01. The heat map and K-means were generated with integrated Differential Expression and Pathway analysis (iDEP) <http://bioinformatics.sdstate.edu/idep93/>. The Kyoto Encyclopedia of Genes and Genomes (KEGG) analysis was performed by DAVID using the DEGs as inputs to assess enriched pathways.

### Statistical analysis

Results were expressed as mean ± standard division of at least three independent experiments. The two-tailed t-test was used to evaluate the significance between means and the *P*-value less than 0.05 was considered statistically significant. Analysis was performed with Graphpad prism 9 (GraphPad Software, SD).

## Results

### The iPSCs survived in the tumors by the co-injection with cancer cells

**Fig. 1:**
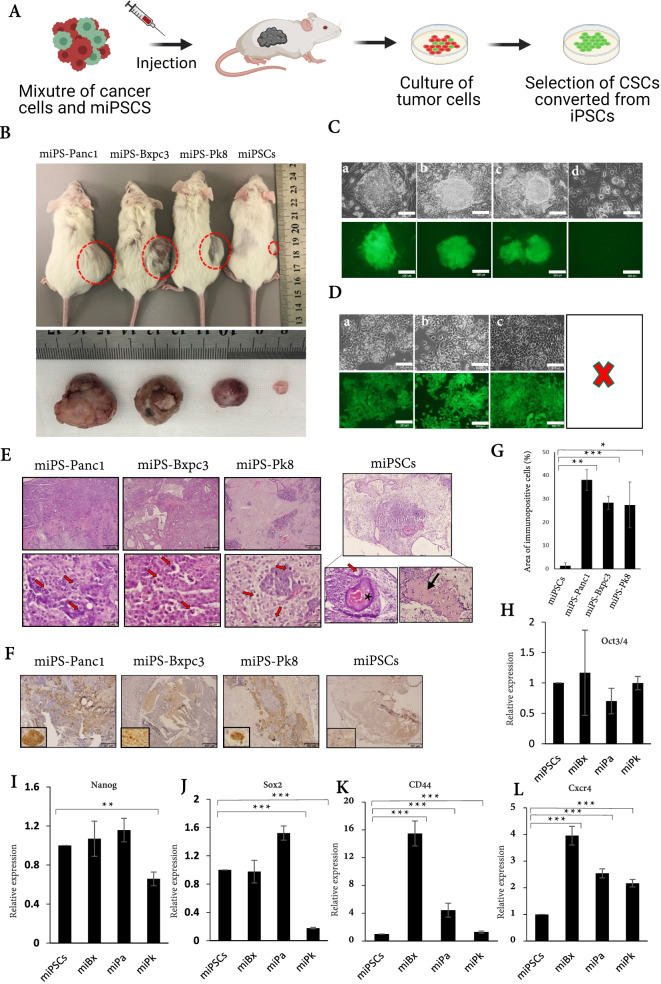
Co-injection of miPSCs with cancer cells maintained the stemness of miPSCs. **A** A schematic design of the experiment. The miPSCs were mixed with human-derived cancer cells, transplanted into mice, and iPSC-derived cells were isolated from the primary culture of the tumors. **B** Representative photographs of tumors in mice and excised from mice. The miPS-Panc1, tumor from mixtures of miPSCs and PANC1 cells; miPS-Bxpc3, tumors from mixtures of miPSCs and BxPC3 cells; miPS-Pk8, tumors from mixtures of miPSCs and PK8 cells. miPSCs denotes the teratoma derived from the injection of only miPSCs. *n* = 3 for each condition. **C** Representative photographs of primary cultures of tumors, miPS-Panc1 cells (**a**), miPS-Bxpc3 cells (**b**), miPS-Pk8 cells (**c**), and miPSCs (**d**), showing colonies of GFP positive cells while the cells from miPSC derived tumors were negative for GFP. **D** Representative photographs of cells survived after the treatment with 1.5 μg/ml of puromycin for 1 week. miPa cells from miPS-Panc1 cells (**a**), miBx from miPS-Bxpc3 cells (**b**), miPk from miPS-Pk8 cells (**c**). The miPSCs were sensitive to this condition and did not survive. **E** The histological stains of tumor sections with hematoxylin and eosin (H&E). The endoderm-derived glands (red arrow), the ectoderm-derived squamous epithelium (asterisk), and the mesoderm-derived cartilage tissues (black arrow) were found in miPSC tumors. No teratoma phenotype but malignant phenotypes with mitotic figures and nuclear atypia (red arrows) were found in miPS-Panc1, miPS-Bxpc3, and miPS-Pk8 tumors. Scale bars = 307 μm (top) and 32 μm (bottom). **F** Immunostaining of the tumor sections with anti-GFP Ab. **G**) A histogram of the area immunoreactive to anti-GFP analyzed by ImageJ software. All data were obtained from three independent tumors (*n* = 3), statistically analyzed, and presented as mean ± SD. Scale bar = 307 μm. **H ~ L** Relative expression levels of stemness and CSC genes assessed by RT-qPCR. All data were obtained from three different experiments and statistically analyzed. *, *p* < 0.05; **, *p* < 0.001; ***, *p* < 0.0001

The origin of CSCs could be hypothesized normal stem cells. Although the factors responsible for the conversion are still controversial, the link between chronic inflammation and cancer initiation could be explored by new models converting stem cells into CSCs. To mimic the existence of stem cells in a highly immunogenic microenvironment enriched with inflammatory, growth factors, and immunogenic responses, we mixed human-derived cancer cells with mouse pluripotent stem cells, miPSCs, and subcutaneously injected them into immunodeficient mice (Fig. 1A). Three different pancreatic cancer cell lines; PK8, PANC1, and BxPC3 cells were chosen and mixed with miPSCs. The miPSCs were designed to express both green fluorescent protein (GFP) and puromycin resistance genes under the control of Nanog promoter so that GFP fluorescent represents undifferentiated state and could be selected with puromycin. Upon differentiation, the cells lose GFP and become sensitive to puromycin. As result, the mixed cells developed tumors with varied sizes between groups and boosted cells’ tumorigenicity where the tumor volumes significantly got bigger than those developed from either cancer cells or miPSCs alone. The mixture of miPSCs and PANC1 cells (miPS-Panc1) exhibited the largest volume followed by the mixture of miPSCs and BxPC3 cells (miPS-Bxpc3) and then that by miPSCs and PK8 cells (miPS-Pk8) (Fig. 1B, Supplementary Fig. 1A). The tumorigenicity was enhanced by the in vivo interaction between iPSCs and cancer cells.

From the tumors, we prepared the primary cultures to investigate the presence of CSCs induced by the impact of mixed microenvironments on miPSCs. We found that the primary culture of mixture tumors had both GFP positive and negative cells in contrast to cells isolated from miPSC tumors which did not show GFP positive cells (Fig. 1C). Cells expressing GFP were selected by treating cells with 1.5 μg/ml of puromycin without leukemia inhibitory factor (LIF) so that the residual miPCs, cancer cells, and host-derived cells should be removed (Fig. 1D). The selected GFP-positive cells were maintained GFP in absence of LIF. Routinely, miPSCs require LIF to maintain stemness in cell culture conditions and begin to differentiate and die when culture without LIF. Also, to confirm the sensitivity of PK8, PANC1, and BxPC3 cells to puromycin, cells were treated with 1.5 μg/ml of puromycin for 4 days, and then the media was replaced with puromycin free media and kept for 2 weeks. After 2 weeks, no cells were survived in the wells of PK8, PANC1, or BxPC3 cells (Supplementary Fig. 1B). Also, flow cytometry analysis showed the efficiency of puromycin to select GFP populations (Supplementary Fig. 1C). Cells isolated from miPSC derived tumors also did not survive when treated with puromycin (Fig. 1D). Accordingly, the GFP positive cells were considered as the converted from miPSCs, isolated from the tumors of miPSCs-Bxpc3, miPSCs-Panc1, and miPSCs-Pk8 and designated as miBx, miPa, and miPK cells, respectively. These cells were confirmed to maintain GFP expression and proliferate without LIF indicating the change of miPSC’s phenotype after co-injection with cancer cells.

The histological analysis of tumors of mixed cells showed malignant phenotypes with mitotic figures and severe nuclear atypia while no normal phenotype was found in those tumors. On the other hand, the sections from miPSC-derived tumors showed benign teratoma phenotype of normal tissue deriving from three germ layers. The endoderm-derived glands, ectoderm-derived squamous epithelium, and mesoderm-derived cartilage tissue were detected in these sections (Fig. 1E). The immunoreactivity to GFP antibody was significantly higher in tumors of mixed cells than that in teratoma (Fig. 1F, G). The presence of GFP should indicate the maintenance of stemness of miPSCs. The immunoreactivity to STEM121, an antibody reacts specifically with a cytoplasmic protein of human cells, showed that more than 95% of tumor sections were of mice origin while only 1 ~ 5% were from human (Supplementary Fig. 1D).

We further analyzed relative expression levels of genes associated with stemness and CSCs in miBx, miPa, and miPk cells compared with those in miPSCs (Fig. 1H, I, J). Oct3/4 expression was maintained in all three cell types. Nanog expression was maintained in miBx and miPa cells but decreased in miPK cells. Sox2 expression was maintained in miBx cells, increased by 1.5-fold in miPa cells, and decreased by 2.5-fold in miPk cells. Expression levels of CSC markers, CD44 and Cxcr4, were increased in miBx, miPa, and miPk cells whereas the highest levels were in miBx cells (Fig. 1K, L).

### Co-injection conferred iPSCs with malignancy and CSC phenotypes

**Fig. 2:**
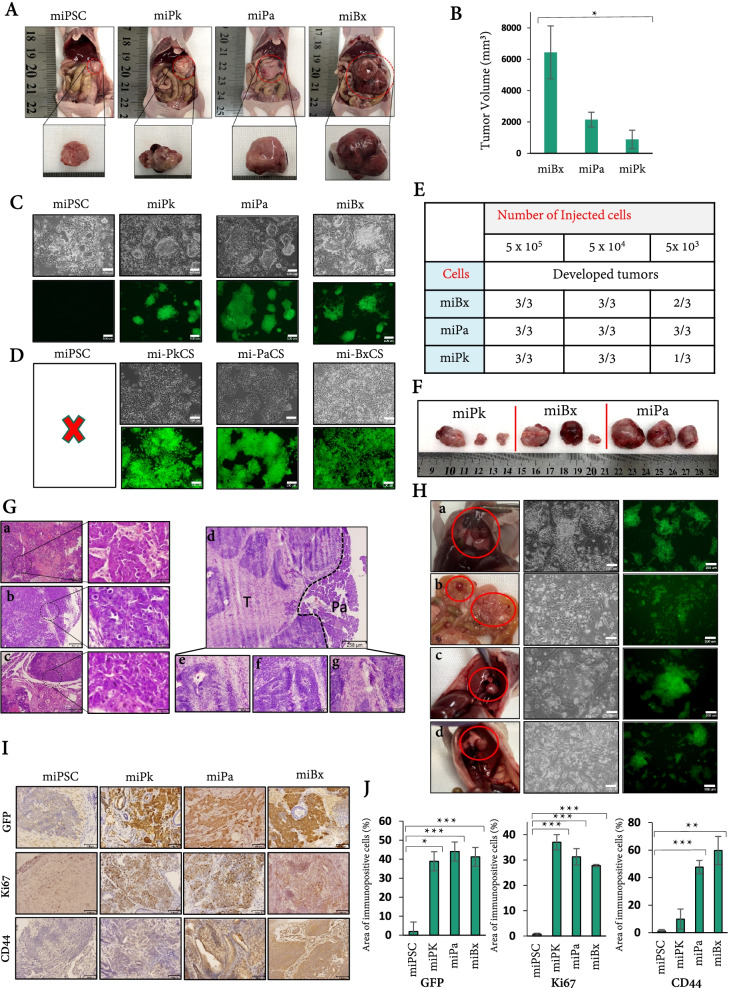
Co-injected miPSCs acquired CSC features. **A** Representative images of mice and isolated tumors resulted from the injection of cells in Fig. 1D. The miBx, miPa, and miPk cells were injected at dose of 5 × 10^5^ cells/mouse and the mice were sacrificed after 4 weeks. Mice injected with miPSCs were used as controls. **B** A bar graph shows the volume of each tumor in **A**. *n* = 3 for each condition. *, *p* < 0.05. **C** Representative images of the primary cultures from tumors formed in the pancreases by miBx, miPa, miPk cells, and miPSCs. Cells were GFP positive forming colonies while those from miPSC tumors were GFP negative. Scale bars = 100 μm. **D** Photographs of the cells in **C** after treatment with 1.5 μg/ml puromycin for 1 week. Scale bars = 100 μm. **E** The summary of tumors formed in the pancreases by miBx, miPa, and miPk cells. Groups of three mice were injected with either 5 × 10^3^, 5 × 10^4^ or 5 × 10^5^ of miBx, miPa or miPk cells/mouse. Mice were sacrificed 4 weeks after injection of 5 × 10^5^ and 5 weeks after injection of 5 × 10^3^ or 5 × 10^4^ cells. **F** Representative images of tumors resulted from the injection of 5 × 10^3^ of miBx, miPa and miPk cells. **G** The histological evaluation of tumors by H&E staining. The tumor sections of miBx (**a**), miPa (**b**), and miPk cells (**c**) showing malignant phenotypes with mitotic figures, nuclear atypia, and high nuclear to cytoplasmic ratio. **d ~ g** Sections of miPSCs tumors showing (**d**) normal pancreatic tissue (Pa) and tumor tissue from the injection of miPSCs (T). The miPSC tumor sections displayed endoderm-derived glands (**e**), ectoderm-derived neuroepithelial tissues (**f**), and mesoderm-derived muscle tissues (**g**). **H** The metastases after injection of cells in the pancreases (left column) and primary cells isolated from the metastases (middle and right columns). Metastasis of miBx cells on the diaphragm (**a**) and the mesentery (**b**), metastasis of miPk cells on the diaphragm (**c**), and metastasis of miPa cells on the diaphragm (**d**). The bright field (middle column), the fluorescence of GFP (left column). **I** Immunostaining of the tumor sections with anti-GFP Ab, anti-Ki67 Ab, and anti-CD44 Ab**.** Scale bars = 64 μm. **J** Histogram of the area immunoreactive to anti-GFP Ab, anti-Ki67 Ab and anti-CD44 Ab. All data were obtained from three independent tumors (*n* = 3), statistically analyzed and presented as mean ± SD. **, *p* < 0.001; ***, *p* < 0.0001

We further investigated the malignant tumorgenicity ability of miBx, miPa, and miPk cells. A half-million of each cell type were injected into the pancreases of Balb/c nude mice, which developed tumors with different aggressiveness. Four weeks after injection, the tumors of miBx cells had the largest volumes and those of miPa cells were the second largest while those of miPk cells were the smallest ones (Fig. 2A, B). Noticeably, sole injection of miPSCs developed small benign tumors. The primary cultures of the tumors derived from miBx, miPa, and miPk cells showed GFP positive cells forming colonies among GFP negative cells in adhesive culture conditions. The cells from the tumors of miPSCs were weakly positive or negative for GFP indicating that they are differentiated cells (Fig. 2C, D). The primary cells from the tumors of miBx, miPa, and miPk cells designated as mi-BxCS, mi-PaCS, and mi-PkCS cells, respectively, were treated with puromycin. The treated cells became homogeneous GFP positive cell populations while cells from tumors of miPSCs did not survive when treated with puromycin (Fig. 2D). The trace of human cancer cells was assessed on the primary cultures by the PCR reaction of genomic DNA to detect sequences of Alu elements, which are primate-specific short-interspersed elements contained in the human genome but not in mice. As result, the primary cells from the tumors of mixed cells (miPSCs-Bxpc3, miPSCs-Panc1, and miPSCs-Pk8 cells) exhibited the presence of human-derived cells, which were eradicated after the selection with puromycin in miBx, miPa, and miPk cells. Moreover, no human genome was detected in the primary cells from the tumors of pancreatic transplants (mi-BxCS, mi-PaCS, and mi-PkCS cells) although they were not treated with puromycin. DNA from human and mouse cells were used as positive and negative controls (Supplementary Fig. 2A). Thus, the tumors developed in the pancreases were of mice origin. The further subcutaneous injections of mi-BxCS, mi-PaCS, and mi-PkCS cells also gave tumors in nude mice confirming sustained tumorigenicity in serial injections (Supplementary Fig. 2B).

The capability of a small number of cells to form a malignant tumor is one of the critical features to define a CSC [21]. Since 5 × 10^5^ cells gave tumors in all injected mice, we further assessed the difference in tumorigenicity of miBx, miPa, and miPk cells injecting 5 × 10^3^ and 5 × 10^4^ cells into the pancreas of nude mice. Injection of 5 × 10^4^ cells also gave rise to tumors in all mice for all three types of cells. When 5 × 10^3^ miPa cells were injected, three in three mice developed tumors while only two in three developed tumors in the case of miBx cells and one in three of miPk cells (Fig. 2E, F). These data suggest that iPSCs could be converted into CSCs with different tumorigenic phenotypes depending on cancer-inducing microenvironments.

The tumors in the pancreases developed from miBx, miPa, and miPk cells including miPSCs were subjected to histological analysis. The sections of miBx tumors (Fig. 2Ga), miPa tumors (Fig. 2Gb), and miPk tumors (Fig. 2Gc) showed malignant phenotypes with mitotic figures, nuclear atypia, and high nuclear to cytoplasmic ratio while those of the tumors developed from miPSCs showed benign phenotypes derived from three germ layers, namely endoderm-derived glands, ectoderm-derived neuroepithelial tissues, and mesoderm-derived muscle tissues (Fig. 2Gd, e, f, g). The miBx and miPa tumors also exhibited undifferentiated tumor phenotype unlike miPk tumors, which exhibited differentiated phenotype with gland-like structures (Supplementary Fig. 2C). The immunohistochemistry staining showed immunoreactivity to keratin 20 (CK20) in the sections of tumors from miBx, miPa, and miPk cells while only miPk tumor sections were immunoreactive to keratin 7 (CK7). The miPSC sections were negative for CK20 and CK7 (Supplementary Fig. 2D).

The sections of miBx, miPa, and miPk cell tumors showed the immunoreactivity to anti-GFP antibody indicating the maintenance of stemness. The immunoreactivities to anti-GFP antibody in miBx, miPa, and miPk sections were significantly higher than that in sections of miPSC tumors. The immunohistochemistry also showed that miBx and miPa tumors were positive for CD44, a CSC marker. The staining with anti-CD44 antibody was stronger in the miBx tumor sections than in those of miPa and miPk tumors while the staining was almost equivalent in the sections of miPk tumors and miPSCs teratoma (Fig. 2I, J).

**Fig. 3:**
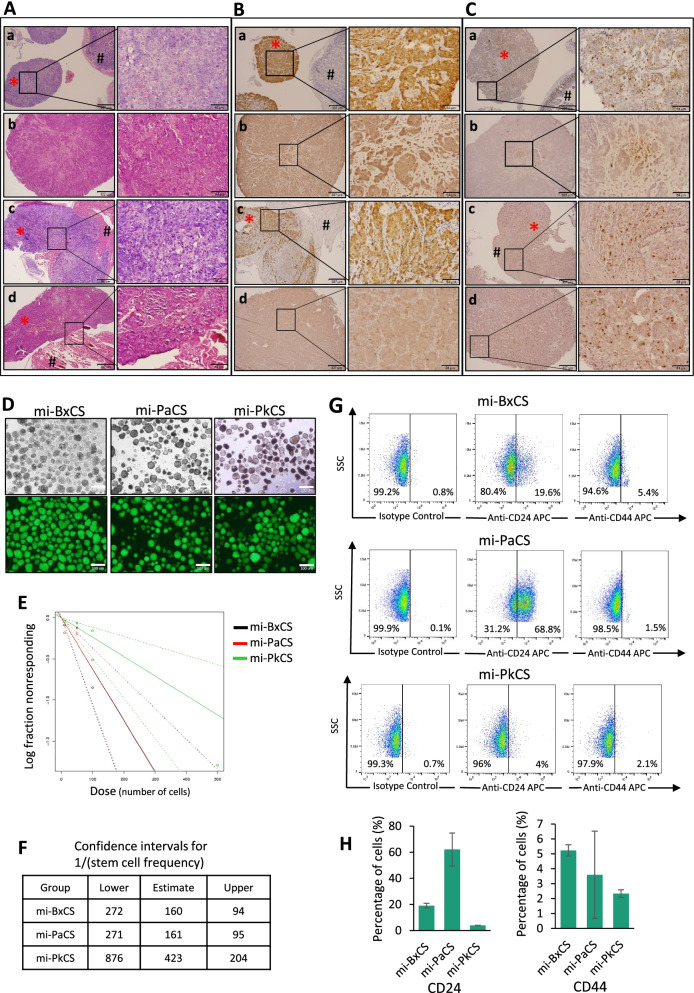
The metastatic and self-renewal potentials and CSC markers in CSC-derived from miPSCs. **A** The histological evaluation of metastases by H&E staining. Sections of mesentery metastasis of miBx cells (**a**), diaphragm metastasis of miBx cells (**b**), diaphragm metastasis of miPk cells (**c**), and sections of diaphragm metastasis of miPa cells (**d**). **B, C** Immunostaining of the metastases with anti-GFP Ab (**B**) and anti-Ki67 Ab (**C**). Sections of mesentery metastasis of miBx cells (**a**), diaphragm metastasis of miBx cells (**b**), sections of diaphragm metastasis of miPk cells (**c**), and sections of diaphragm metastasis of miPa cells (**d**). Asterisks show metastases, Hashes show normal tissues. Scale bars =307 μm (left column) and 64 μm (right column). **D** Representative bright field and fluorescent images of spheroid formed by mi-BxCS, mi-PaCS, and mi-PkCS cells after 1 week of culturing in low adherent conditions with free serum media. Scale bars = 100 μm. **E** Sphere-forming potential of mi-BxCS, mi-PaCS, and mi-PkCS cells assessed by ELDA. **F** Summary of the frequency of CSCs estimated by the ELDA. Confidence choice was 0.95. **G** Representative flow cytometry plots of mi-BxCS, mi-PaCS, and mi-PkCS cells assessed by anti-CD24 Ab and anti-CD44 Ab. *n* = 3. **H** Bar graphs showing the percentage of cells positive to CD24 and CD44 for each cell type. Data were from three independent experiments of flow cytometry. *n* = 3

When injected into the pancreases, miBx, miPa, and miPk cells exhibited different metastatic abilities. The metastasis of miBx cells was remarkable showing nodules on the diaphragm and the mesentery while the metastasis of both miPa and miPk cells was observed only on the diaphragm (Fig. 2H). The primary cultures from all the tumors of metastasis showed both GFP positive and negative cells confirming that injected cells into the pancreases metastasized to these locations (Fig. 2H). On the other hand, injection of miPSCs did not show any metastases. The sections from metastases showed malignant phenotypes and immunoreactivity to anti-GFP and anti-Ki67 antibodies (Fig. 3A,B,C). Collectively, the malignant phenotypes of our novel cells were confirmed with the characters of CSCs.

### CSC-derived from iPSCs exhibited self-renewal, differentiation, and migration characteristics and different drug responses

The CSCs are often characterized by their ability of self-renewal as spheroids in serum-free media as well as the growth in anchorage-independent conditions [22]. When cultured in low-adherent dishes with serum-free media, mi-BxCS, mi-PaCS, and mi-PkCS cells formed spheroids with GFP expression (Fig. 3D). To further assess the difference in self-renewal capacity between these cells, we performed the extreme limiting dilution assay (ELDA) which is used to determine the frequency of cancer-initiating cells in a cell population. The estimated tumor-initiating cell frequencies with 95% confidence intervals (CI) were found to be 160, 161, and 423 for mi-BxCS, mi-PaCS, and mi-PkCS cells, respectively (Fig. 3E, F). These data are also consistent with in vivo one which showed that miBx and miPa cells are more tumorigenic than miPk cells (Fig. 2E, F). The flow cytometric analysis also showed that the percentage of cells positive to CD24 or CD44 were higher in mi-BxCS and mi-PaCS cells than that in mi-PkCS cells (Fig. 3G, H). Approximately 60, 20 and 5% of mi-PaCS, mi-BxCS and mi-PkCS cells were CD24 positive, respectively, while 3, 5 and 2% of respective cells were CD44 positive.

**Fig. 4:**
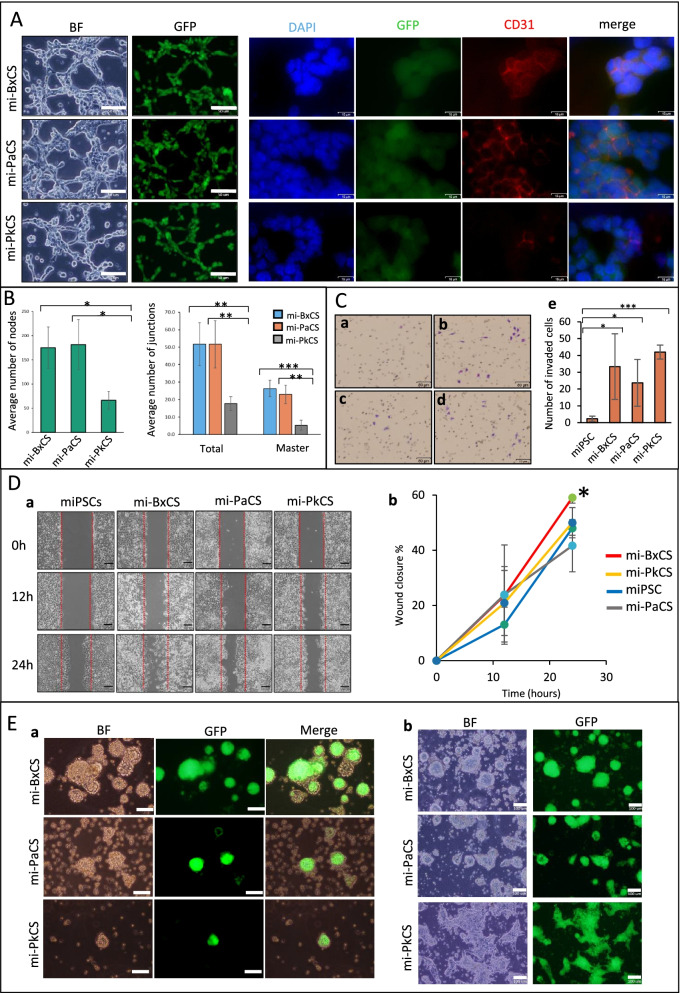
Differentiation, invasion, and migration characteristics of CSCs converted from miPSCs. **A** Representative images of bright field and fluorescence of tube structures (Scale bars = 100 μm) and immunofluorescence of cells stained with anti-CD31 Ab (Scale bars = 16 μm). Tube formation assay was performed where cells were cultured on Matrigel for 24 h. **B** The average number of nodes, total junctions, and master junctions per field in tube forming structures formed by mi-BxCS, mi-PaCS, and mi-PkCS cells. Data were obtained from three independent experiments and analyzed by ImageJ. *n* = 3; *, *p* < 0.05; **, *p* < 0.001. **C** Representative images of invasion assay. miPSCs (**a**), mi-BxCS (**b**), mi-PaCS (**c**), and mi-PkCS (**d**) cells. Scale bars =80 μm, a bar graph of the average number of invaded cells (**e**). *, *p* < 0.05; ***, *p* < 0.0001. **D** Scratch assay, representative images of cells at 12 and 24 h after scratching (**a**). Scale bars = 100 μm, a graph presenting the percentage of wound closure at the indicated time points (**b**). *, *p* < 0.05. **E** The 3D structures showing GFP positive and negative structures, floating structures (**a**) and adherent colonies (**b**). Scale bar =80 μm for (**a**) and 100 μm for (**b**)

Cancer cells can promote tumor angiogenesis in different ways of vascular tube formation such as vascular mimicry and induction of endothelial cells by differentiation of other types of cells [23]. In previous reports, we showed that CSCs could differentiate into CD31 positive cells with the ability to form tube structures in the presence of type IV collagen [24, 25]. When cultured on Matrigel, mi-BxCS, mi-PaCS, and mi-PkCS cells formed tube-like structures without exogenous angiogenic factors (Fig. 4A). The mi-BxCS and mi-PaCS cells were found to have more potential to form tube structures where the average numbers of nodes and junctions formed by mi-BxCS and mi-PaCS cells were significantly higher than those formed by mi-PkCS cells (Fig. 4B). All three types of cells also exhibited an enhanced ability of invasion when assessed by trans-well invasion assay. The number of invaded cells was significantly higher in mi-BxCS, mi-PaCS, and mi-PkCS cells than that in miPSCs (Fig. 4C). Applying the scratch assay, we compared the mobility of mi-BxCS, mi-PaCS, and mi-PkCS cells. The mobility of mi-BxCS, mi-PaCS, and mi-PkCS cells was higher than that of miPSCs. Within 12 h, 24% of the scratch area was healed by the migration of mi-BxCS and mi-PaCS cells and 21% healed by mi-PkCS cells while only 13% healed by miPSCs. After 24 h, the percentage of the closed area became 32, 59, 42, and 50% for miPSCs, mi-BxCS, mi-PaCS, and mi-PkCS cells, respectively (Fig. 4D).

The cancer cell heterogeneity is explained by the hierarchical model of CSCs where CSCs differentiate into heterogeneous phenotypes [24, 26]. As we have shown above, mi-BxCS, mi-PaCS, and mi-PkCS cells formed spheroids maintaining the stemness as well as GFP expression in serum-free media and low adherent conditions. The mi-BxCS, mi-PaCS, and mi-PkCS cells also exhibited different morphologies in adherent conditions. While mi-BxCS and mi-PkCS cells had mesenchymal-like morphology prone to spread, mi-PaCS had epithelial-like morphology to form compact colonies attached to the dishes (Supplementary Fig. 3A).

The GFP-positive cells were further evaluated for the ability of differentiation in non-coated Petri dishes considering as 3D culture of semi-adherent condition. The mi-BxCS, mi-PaCS, and mi-PkCS cells were cultured in dishes with serum-free media supplemented with EGF and FGF2. The mi-BxCS and mi-PaCS cells formed organoids of floating 3D structures while some cells formed colonies attached to the dishes. The organoid-like structures of mi-BxCS showed both GFP positive and negative parts. The mi-PkCS cells were found to have more adhesive characters where a very small portion of cells formed 3D structure shown to be GFP positive and a majority of mi-PkCS cells was adherent (Fig. 4E). When organoids of floating 3D structures were cultured on gelatin-coated dishes with serum-supplemented media, they gave attached differentiated cells as GFP negative cells (Supplementary Fig. 3B). Collectively, the 3D structures apparently constituted heterogeneous structures mimicking tumor tissues as cancer organoids.

Finally, we assessed the efficacy of two different anticancer drugs, gemcitabine and doxorubicin, on mi-BxCS, mi-PaCS, and mi-PkCS cells. The cells showed different responses to gemcitabine, a nucleotide analogue, and doxorubicin, a topoisomerase II inhibitor. As for doxorubicin, mi-PkCS cells were more sensitive to show IC_50_ at 50 nM than mi-BxCS and mi-PaCS cells showing IC_50_s at 110 nM and 75 nM, respectively while Balb/c 3 T3 cells were resistant with an IC_50_ of 1.9 μM. Gemcitabine showed almost similar IC_50_s on mi-BxCS, mi-PkCS, mi-PaCS and Balb/c 3 T3 cells at 375 nM, 490 nM, 530 nM and 505 nM, respectively (Supplementary Fig. 4). With the IC_50_ ranging from 12 to 50 nM on pancreatic cancer cell lines; BxPC3 and PANC-1, gemcitabine is accepted as the first-line chemotherapeutic drug for the treatment of pancreatic cancer. However, the IC_50_s assessed in this study were approximately 10 folds higher than that reported in previous studies [27].

### Co-injected iPSCs enrich cancer-related pathways and changing metabolic ones

**Fig. 5:**
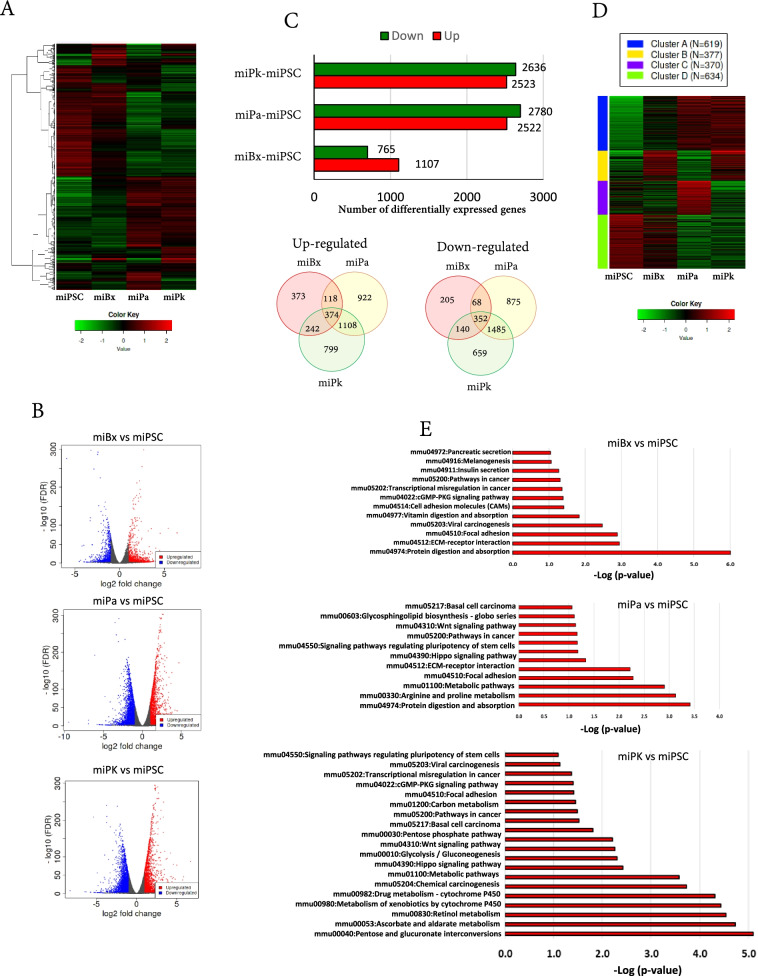
Analysis of the transcriptome of CSCs converted from miPSCs by RNA-seq. **A** A heat map of expression patterns of miPSCs and CSCs converted from miPSCs. **B** Volcano plots of differentially expressed genes in CSCs converted from miPSCs compared with miPSCs showing up- and downregulating genes. **C** A graph bar for the number of up- and downregulating genes in CSCs compared with miPSCs and Venn diagrams show the differentially up- and downregulating genes in three types of CSCs while each of them was compared with miPSCs. **D** A heat map of K-means clustering and enrichment analysis. **E** The top enriched pathways for the upregulated genes in CSCs converted from miPSCs compared with miPSCs

We performed RNA-sequencing on miBx, miPa, miPk cells and miPSCs and compared the expression patterns of these cells as a heat map (Fig. 5A). The differentially expressed genes (DEGs) were summarized as volcano plots (Fig. 5B). With a false discovery rate (FDR) < 0.01, a total of 5159, 5302, and 1872 DEGs were identified for miPk, miPa, and miBx cells versus miPSCs, respectively (Fig. 5C). The number of upregulated genes were 2523, 2522, and 1107, while the number of downregulated ones were 2636, 2780, and 765 for miPk, miPa, and miBx cells, respectively (Fig. 5C). Gene ontology and biological pathway analyses showed a significant enrichment of pathways and biological processes related to cancer, stemness, and metabolism (Fig. 5D, E) and (Supplementary Table 1). The KEGG pathway enrichment analysis of DEGs showed that miBx, miPa, and miPk cells had common enriched pathways including the entries “mmu04510: Focal adhesion”, “mmu04974: Protein digestion and absorption” and “mmu05034: Alcoholism” (Supplementary Table 2) and (Fig. 5E). We further investigated the list of DEGs in the three common pathways and found the most significant changing genes among pathways were Actn3, Ccnd2, Col4a1, Col4a2 (Supplementary Table 3, Supplementary Table 4).

Next, we focused on metabolic pathways enriched in either miBx, miPa, or miPk cells and further investigated the expression levels of genes of those pathways which identified by KEGG analysis (Supplementary Table 2) by extracting the expression count from normalized expression count (Supplementary Table 3). Importantly, we found that the expression levels of 20 genes related to metabolism were significantly upregulated in the converted cells compared with those in miPSCs (Supplementary Table 4). We repeated the analysis of KEGG on the 20 genes and found that the pathway enriched with those genes included two groups of genes which were common between those pathways. Seven genes were Lhpp, Ndufa2, Ndufa3, Ndufa4l2, Ndufs3, Ndufs6, and Ndufv3 in the entry “mmu00190: Oxidative phosphorylation”. Five genes were Pfkl, Pfkm, Pgam1, Pgm2, and Tpi1 in the entry “mmu00010: Glycolysis /Gluconeogenesis” (Supplementary Table 5). Looking into the expression count data of these genes, high count numbers with significantly different from the expression in miPSCs narrowed the genes to Ndufv3, Ndufa2, Ndufs6, Ndufa4l2, and Ndufa3 in the entry “mmu00190”, and Tpi1, Pfkl, Pgm2, and Pgam1 in the entry “mmu00010” pathways (Supplementary Table 4). The expression count data also showed that CSCs are expressing different isoforms of aldehyde dehydrogenase (ALDH) and among those isoforms, Aldh18a1, Aldh16a1, Aldh1b1 and Aldh1l1 had the highest expression values with variation between cell type (Supplementary Table 4).

Since converted cells exhibited different responses to anticancer drugs, we investigated the expression levels of multidrug resistance (MDR) genes which are responsible for drug resistance. From the expression count data, the expressions of Abcb1b, Abcg2, Abcc4, and Abca1 were found to be at highest levels in mi-BxCS cells and lowest in mi-PkCS cells while they had intermediate levels in mi-PaCS cells (Supplementary Table 6).

## Discussion

Here we demonstrated an in vivo method for conversion of normal stem cells, miPSCs, into CSCs and showed cancerous microenvironment effective on stem cells could be artificially realized in vivo by mixing cancer cells with iPSCs to confer iPSCs with malignant phenotype. Notably, the tumorigenicity of miPSCs in mixtures was significantly boosted and varied depending on three types of cancer cells mixed with miPSCs. Conceivably, three different cancer cell lines used in this study apparently provided their specific secretome and cell surface microenvironments to miPSCs resulting in the conversion of cells into various phenotypes. The common properties of CSCs obtained from iPSCs were the potentials of migration, invasion, and malignancy, which were not always equivalent between the CSCs notwithstanding of the same origin. As a result, two different cell morphologies were noticed in the converted CSCs, mesenchymal-like morphology for miBx and miPk cells and epithelial-like morphology for miPa cells. The BxPC3 and PANC1 cells are the effector cancer cell lines derived from pancreas origin while PK8 cells are the ones from a liver metastasis of pancreatic origin. This difference of characters could be responsible for the difference in tumorigenicity of the CSCs. As we noticed that CSCs resulted from the mixture of PK8 cells with miPSCs were less tumorigenic when compared with those from the mixture of either BxPC3 or PANC1 cells.

The concept that epigenetic changes by inflammation affecting cancer induction is well accepted but the cell types affected by inflammation are still controversial [1, 4, 28]. We are hypothesizing that the cell types should be undifferentiated progenitor cells with pluripotent characters localized in every tissue. Previously, we have shown in different reports that conditioned media from different cancer cell line cells can convert iPSCs into CSCs. In those reports, cells were cultured in the presence of CM for almost one month to complete conversion [18–20, 29]. To establish a more efficient way of conversion, which integrates cell-cell interactions between different types of cells and iPSCs, we tried to expose iPSCs to cancer cells in vivo, which would be a more real and spontaneous condition of inflammation than in vitro including the immune responses at the same time. Our data support the insight into the induction of cancer, which could be enhanced by the homeostasis imbalanced for a long period converting stem cells into CSCs, and the development of tumors acquiring metastatic potential.

Interestingly, the response to drugs was different between miBx, miPa, and miPk cells even they were originated from same cells, miPSCs. The IC_50_s for gemcitabine was approximately 10 folds higher than that reported in previous studies while doxorubicin showed IC_50_s on these cells at around 50 to 100 nM, which is feasibly low to be a candidate as drug targeting CSCs [27]. This result is consistent with our previous report [30]. These differences could be traced to the mechanism of action of the two drugs, of which efficacy is depending on the cell phenotypes. The difference should be elucidated by the different plasticity in the CSC features, which was conferred to the original iPSCs from the different cancer cells. Gene expression profiles from RNA sequencing of the CSCs showed different levels of expression of ABC transporter family genes responsible for drug resistance. From the profiles, the expressions of Abcb1b, Abcg2, Abcc4, and Abca1 had the highest level in mi-BxCS cells and the lowest in mi-PkCS cells while they had intermediate levels in mi-PaCS cells. The expression levels were appeared consistent with the resistance to gemcitabine and doxorubicin since both of the drugs should be ejected outside of the cells by MDR. Although the IC_50_s in our results were high, the CSCs exhibited the sensitivity to doxorubicin elucidating the expression levels of MDRs in each CSC. On the other hand, the CSCs were more resistant to gemcitabine than doxorubicin showing nearly the same levels of IC_50_s in three types of CSCs converted from iPSCs. This is probably because gemcitabine has to compete with abundant nucleoside molecules in cells to be integrated into DNA and moreover is being transferred from the outside of cells [31]. Ejection of gemcitabine by MDR might be effective enough to show the insensitivity while the doxorubicin acts as a topoisomerase inhibitor preventing the DNA replication which has a small number of competitors when compared to nucleosides [32]. Therefore, doxorubicin, exhibiting sensitivity depending on the expression of MDR molecules, was shown to be more effective than gemcitabine. The difference in the MDR between miBx, miPa, and miPk cells could be due to the different phenotypes. Stem cell plasticity refers to the ability to give other cell phenotypes. The more plastic the stem cells are, the more phenotypes will appear. The plasticity of CSCs is usually defined as the ability to switch between CSCs and differentiated cancer cells. Since CSCs could have different stages and directions of plasticity, different CSCs such as miBx, miPa, and miPk cells may variously respond to drugs.

The search for the upregulated genes in three types of CSCs revealed Actn3, Ccnd2, Col4a1, and Col4a2, which have been linked to tumor aggressiveness, poor prognosis, cancer cell proliferation, and cell migration [33–38]. Another key finding through KEGG pathway analysis of the gene expression profiles indicated changes in metabolic pathways. Comparing the profiles, the genes related to oxidative phosphorylation and glycolysis /gluconeogenesis were found upregulated in all CSCs converted from iPSCs. More specifically, Ndufv3, Ndufa2, Ndufs6, Ndufa4l2, Ndufa3, Tpi1, Pfkl, Pgm2, and Pgam1 genes were found to be the most significant genes. The Ndufv3, Ndufa2, Ndufs6, Ndufa4l2, and Ndufa3 are the components of the mitochondrial enzyme that oxidizes NADH to NAD supporting ATP synthesis. Tpi1, Pfkl, Pgm2, and Pgam1 are the enzymes associated with glycolysis, which is also essential for energy production. These genes have independently been described to have essential roles in carcinogenesis, tumor growth, invasion, and metastasis in different types of cancers regulating glycolysis [39–43]. The shift of metabolism from glycolysis to oxidative phosphorylation or vice versa in CSCs has been described in many reports [44–46] . On the other hand, oxidative phosphorylation was shown to be increased in mesenchymal stem cells transformed with genetic modulation [47]. Some other reports also showed that disorders in metabolite profile could initiate tumor through reprogramming of adult stem cell metabolism [48–51]. Our data also point to the notion of a compensatory system in CSCs where they can shift between glycolysis and oxidative phosphorylation. Collectively, the abnormal microenvironment changes the metabolism of iPSCs converting them into malignant cells with a new energy acquisition mode. In our in vivo study, the cancerous microenvironment might enforce iPSCs, which are metabolically plastic enough, to rewire the metabolism of nutrients conferring malignancy.

The iPSC technology has facilitated personalized medicine and disease modeling [13–15]. Our method as well as the in vitro methods we have been working on could provide tools available for the establishment of both tumor models and precision medicine. The availability of iPSC in this technology offers a unique advantage to provide CSC models for individuals who have risks of cancer even before cancer develops. In this context, iPSCs could be invested to create specific CSC models for each individual. The CSCs derived from patient’s iPSCs could use genomic, transcriptomic, proteomic, and metabolomic analyzing tools and enable screening and prediction of effective treatments for different types of cancers depending on personal genetic and epigenetics profiles.

## Conclusions

Our study and model provide insights into how the microenvironment disordered in vivo converts normal stem cells into CSCs rewiring cell metabolism. This conceptual framework could provide CSC models available for us to take the CSC plasticity into consideration in the studies of CSC induction and the development of therapeutic strategy. Further investigation will be required to expand our knowledge about the role of metabolic pathway shifting in CSCs when they generate.

## Supplementary Information

**Additional file 1: Supplementary Table 1**

**Additional file 2: Supplementary Table 2**

**Additional file 3: Supplementary Table 3**

**Additional file 4: Supplementary Table 5**

**Additional file 5: Supplementary Figure 1.** A) Bar graphs of the volume of tumors derived from BxPC3, PANC1, PK8, miPSCs, mixtures of miPSCs and PANC1 cells (miPS-Panc1), miPSCs and BxPC3 cells (miPS-Bxpc3), and miPSCs and PK8 cells (miPS-Pk8) subcutaneously injected into NOD-SCID mice. *n* = 3 for each condition. *, *p* < 0.05; **, *p* < 0.001. B) Representative images of BxPC3, PANC1, and PK8 cells cultured in 12 well plates and treated with 1.5 μg/ml of puromycin for 4 days and then the media was replaced to media without puromycin and kept for 2 weeks. No survived cells were detected after 2 weeks. C) Representative flow cytometry plots of miBx, miPa, and miPk cells showing GFP positive cell population after selection with puromycin. D) Immunostaining of the tumor sections with STEM121 Ab (a), a bar graph of the area immunoreactive to STEM121 Ab analyzed by ImageJ software (b), and a bar graph of average of percentage of section area of mice or human origin (c). Data were obtained from three independent tumors (*n* = 3), statistically analyzed and presented as mean ± SD. Scale bar = 155 μm. **Supplementary Figure 2.** A) Agarose gel electrophoresis of DNA amplified by PCR with human specific primers, Alu primers. Cells lanes 1,2, and 3 for miPS-Bxpc3, miPS-Panc1, and miPS-Pk8 cells, respectively, which are cells before puromycin treatment. Lanes 4,5, and 6 for miBX, miPa, and miPk cells respectively, which are cells after puromycin treatment. Lanes 7,8, and 9 for mi-BxCS, mi-PaCS, and mi-PkCS cells which are primary cultures form pancreatic tumors without puromycin treatment. Lane 10; BxPC3 cells as positive control. Lane 11: miPSCs as negative control. B) Representative images of tumors from miPSCs, mi-BxCS, mi-PaCS, and mi-PkCS cells. Cells, 1 × 10^5^ cells, were subcutaneously injected into nude mice. *n* = 3 for each cell type. C) The histological evaluation of tumors by H&E staining. The tumor sections of miBx, miPa, and miPk cells showing undifferentiated phenotype for miBx and miPa cell tumors and gland like structures (arrows) in tumors of miPk cells. Scale bars = 40 μm. D) Immunostaining of the tumor sections with anti-CK Ab, anti-CK20 Ab. Scale bars = 32 μm. **Supplementary Figure 3.** A) Representative images of bright field and fluorescence of mi-BxCS, mi-PaCS, and mi-PkCS cells showing differences in cell morphology. Objective lenses 20X and 40X. The miBX and miPk exhibited mesenchymal like morphology while miPa had epithelial like morphology with compact colonies. B) Representative images of mi-BxCS, mi-PaCS, and mi-PkCS cells attached to dishes. a) adherent portions of cancer organoid like structures showing GFP positive and negative cells under semi-adherent conditions without serum. Scale bars = 100 μm, b) representative images of organoid like structures under adherent condition with serum. Cells were transferred from (Fig. 4Ea) to gelatin coated dishes. Scale bars = 100 μm. **Supplementary Figure 4.** A) Dose–response curves of mi-BxCS, mi-PaCS, mi-PkCS and Balb/c 3 T3 cells to doxorubicin and gemcitabine. Cells were treated with different concentrations of drugs for 72 h and viability was quantified by MTT after the treatment. Each plot was taken from three independent experiments. Each plot is depicted as means ± SD. B) Bar graphs of the IC_50_s obtained from C) for doxorubicin and gemcitabine in mi-BxCS, mi-PaCS, mi-PkCS and Balb/c 3 T3 cells. **Supplementary Table 4.** Gene expression count numbers of the most significant changing genes among pathways and metabolic related genes. **Supplementary Table 6.** Gene expression count numbers of MDR genes. **Supplementary Table 7.** Antibody information. **Supplementary Table 8.** Primer sets used in the study.

## Data Availability

All data generated or analyzed during this study are included in this published article and its Supplementary files.

## Abbreviations

iPSCs: Induced pluripotent stem cells
CSCs: Cancer stem cells
CM: Conditioned media
GFP: Green fluorescent protein
RT-qPCR: Quantitative reverse transcription
CK7: Keratin 7
CK20: Keratin 20
H&E: Hematoxylin and eosin
FDR: False discovery rate
DEGs: Differentially expressed genes
KEGG: Kyoto encyclopedia of genes and genome
MDR: Multidrug resistance
IC_50_: The half-maximal inhibitory concentration
CO_2_: Carbon dioxide
DAB: 3, 3′-diaminobenzidine
ELDA: Extreme limiting dilution analysis
ITS-X: Insulin-Transferrin-Selenium-Ethanolamine
NEAA: MEM Non-Essential Amino Acids
FGF-2: Fibroblast growth factor-2
EGF: Epidermal growth factor
