# Supplementary material for: Different pancreatic cancer microenvironments convert iPSCs into cancer stem cells exhibiting distinct plasticity with altered gene expression of metabolic pathways
Source: J Exp Clin Cancer Res. 2022 Jan 21;41:29. doi: 10.1186/s13046-021-02167-3 (PMC8781112; doi:10.1186/s13046-021-02167-3)
Supplement: Supplementary file 5 — Additional file 5: Supplementary Figure 1. A) Bar graphs of the volume of tumors derived from BxPC3, PANC1, PK8, miPSCs, mixtures of miPSCs and PANC1 cells (miPS-Panc1), miPSCs and BxPC3 cells (miPS-Bxpc3), and miPSCs and PK8 cells (miPS-Pk8) subcutaneously injected into NOD-SCID mice. n = 3 for each condition. *, p < 0.05; **, p < 0.001. B) Representative images of BxPC3, PANC1, and PK8 cells cultured in 12 well plates and treated with 1.5 μg/ml of puromycin for 4 days and then the media was replaced to media without puromycin and kept for 2 weeks. No survived cells were detected after 2 weeks. C) Representative flow cytometry plots of miBx, miPa, and miPk cells showing GFP positive cell population after selection with puromycin. D) Immunostaining of the tumor sections with STEM121 Ab (a), a bar graph of the area immunoreactive to STEM121 Ab analyzed by ImageJ software (b), and a bar graph of average of percentage of section area of mice or human origin (c). Data were obtained from three independent tumors (n = 3), statistically analyzed and presented as mean ± SD. Scale bar = 155 μm. Supplementary Figure 2. A) Agarose gel electrophoresis of DNA amplified by PCR with human specific primers, Alu primers. Cells lanes 1,2, and 3 for miPS-Bxpc3, miPS-Panc1, and miPS-Pk8 cells, respectively, which are cells before puromycin treatment. Lanes 4,5, and 6 for miBX, miPa, and miPk cells respectively, which are cells after puromycin treatment. Lanes 7,8, and 9 for mi-BxCS, mi-PaCS, and mi-PkCS cells which are primary cultures form pancreatic tumors without puromycin treatment. Lane 10; BxPC3 cells as positive control. Lane 11: miPSCs as negative control. B) Representative images of tumors from miPSCs, mi-BxCS, mi-PaCS, and mi-PkCS cells. Cells, 1 × 10^5^ cells, were subcutaneously injected into nude mice. n = 3 for each cell type. C) The histological evaluation of tumors by H&E staining. The tumor sections of miBx, miPa, and miPk cells showing undifferentiated phenotype for miB [file 13046_2021_2167_MOESM5_ESM.docx]

**Supplementary information**

**Different pancreatic cancer microenvironments convert iPSCs into cancer stem cells exhibiting distinct plasticity with altered gene expression of metabolic pathways**


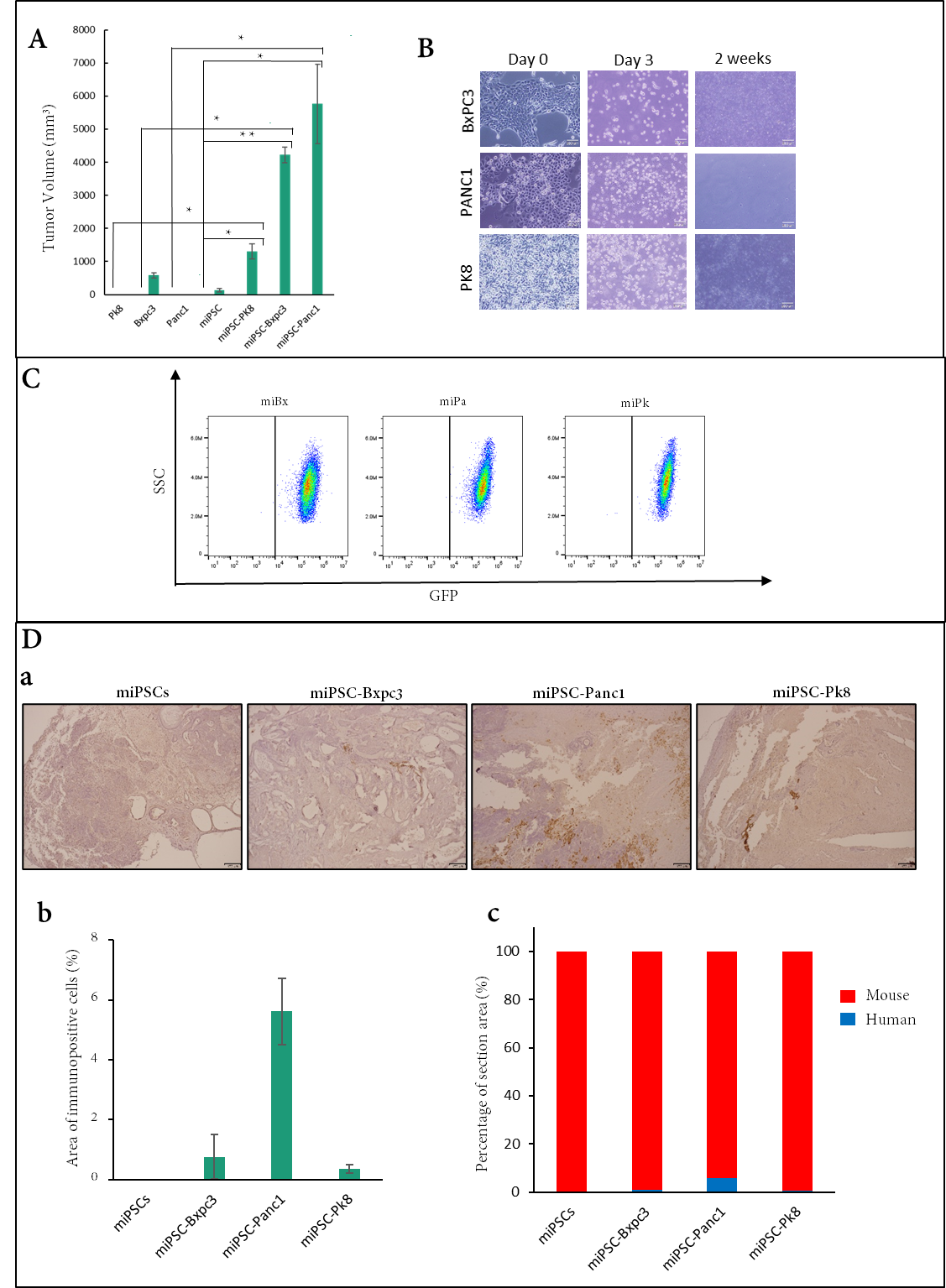


**Supplementary Figure 1.**

**A)** Bar graphs of the volume of tumors derived from BxPC3, PANC1, PK8, miPSCs, mixtures of miPSCs and PANC1 cells (miPS-Panc1), miPSCs and BxPC3 cells (miPS-Bxpc3), and miPSCs and PK8 cells (miPS-Pk8) subcutaneously injected into NOD-SCID mice. n=3 for each condition. *, p < 0.05; **, p < 0.001. **B)** Representative images of BxPC3, PANC1, and PK8 cells cultured in 12 well plates and treated with 1.5 μg/ml of puromycin for 4 days and then the media was replaced to media without puromycin and kept for two weeks. No survived cells were detected after two weeks. **C)** Representative flow cytometry plots of miBx, miPa, and miPk cells showing GFP positive cell population after selection with puromycin. **D)** Immunostaining of the tumor sections with STEM121 Ab (a), a bar graph of the area immunoreactive to STEM121 Ab analyzed by ImageJ software (b), and a bar graph of average of percentage of section area of mice or human origin (c). Data were obtained from three independent tumors (n = 3), statistically analyzed and presented as mean ± SD. Scale bar=155 μm.


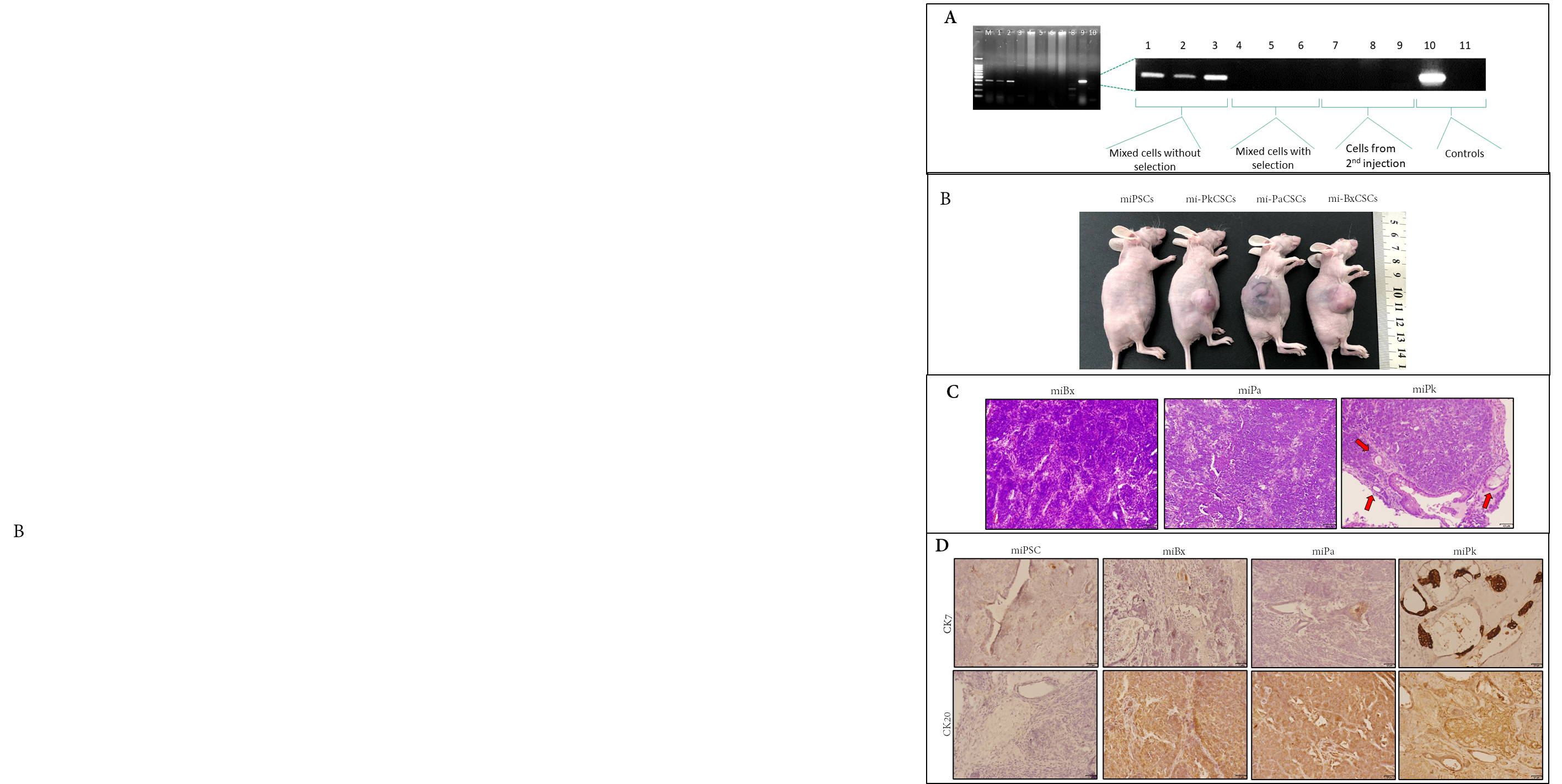


**Supplementary Figure 2.**

**A)** Agarose gel electrophoresis of DNA amplified by PCR with human specific primers, Alu primers. Cells lanes 1,2, and 3 for miPS-Bxpc3, miPS-Panc1, and miPS-Pk8 cells, respectively, which are cells before puromycin treatment. Lanes 4,5, and 6 for miBX, miPa, and miPk cells respectively, which are cells after puromycin treatment. Lanes 7,8, and 9 for mi-BxCS, mi-PaCS, and mi-PkCS cells which are primary cultures form pancreatic tumors without puromycin treatment. Lane 10; BxPC3 cells as positive control. Lane 11: miPSCs as negative control. **B)** Representative images of tumors from miPSCs, mi-BxCS, mi-PaCS, and mi-PkCS cells. Cells, 1 × 10^5^ cells, were subcutaneously injected into nude mice. n=3 for each cell type. **C)** The histological evaluation of tumors by H&E staining. The tumor sections of miBx, miPa, and miPk cells showing undifferentiated phenotype for miBx and miPa cell tumors and gland like structures (arrows) in tumors of miPk cells. Scale bars=40 μm. **D)** Immunostaining of the tumor sections with anti-CK Ab, anti-CK20 Ab**.** Scale bars= 32 μm.


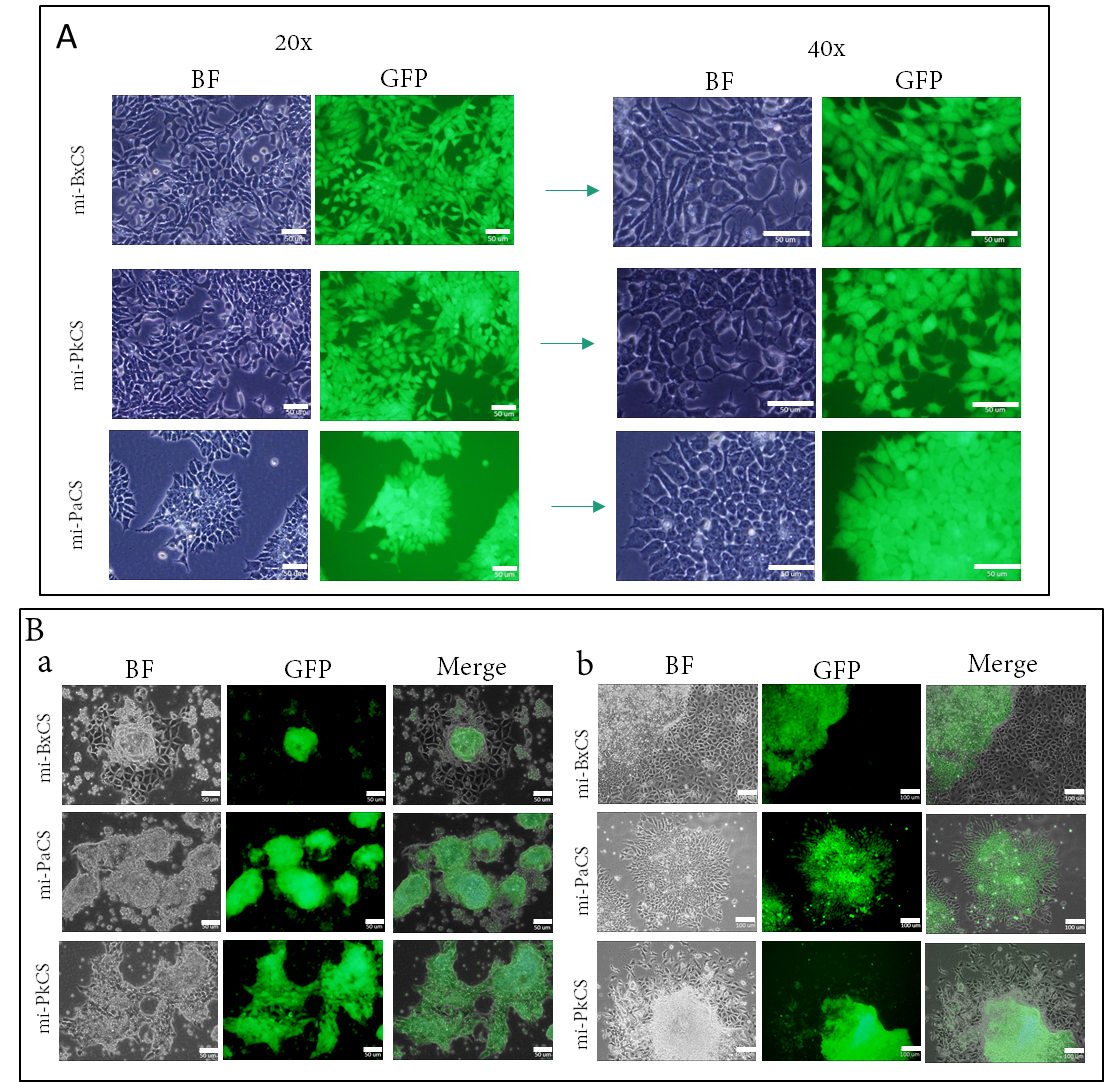


**Supplementary Figure 3.**

**A)** Representative images of bright field and fluorescence of mi-BxCS, mi-PaCS, and mi-PkCS cells showing differences in cell morphology. Objective lenses 20X and 40X. The miBX and miPk exhibited mesenchymal like morphology while miPa had epithelial like morphology with compact colonies. **B)** Representative images of mi-BxCS, mi-PaCS, and mi-PkCS cells attached to dishes. a) adherent portions of cancer organoid like structures showing GFP positive and negative cells under semi-adherent conditions without serum. Scale bars = 100μm, b) representative images of organoid like structures under adherent condition with serum. Cells were transferred from (figure 4Ea) to gelatin coated dishes. Scale bars = 100μm.


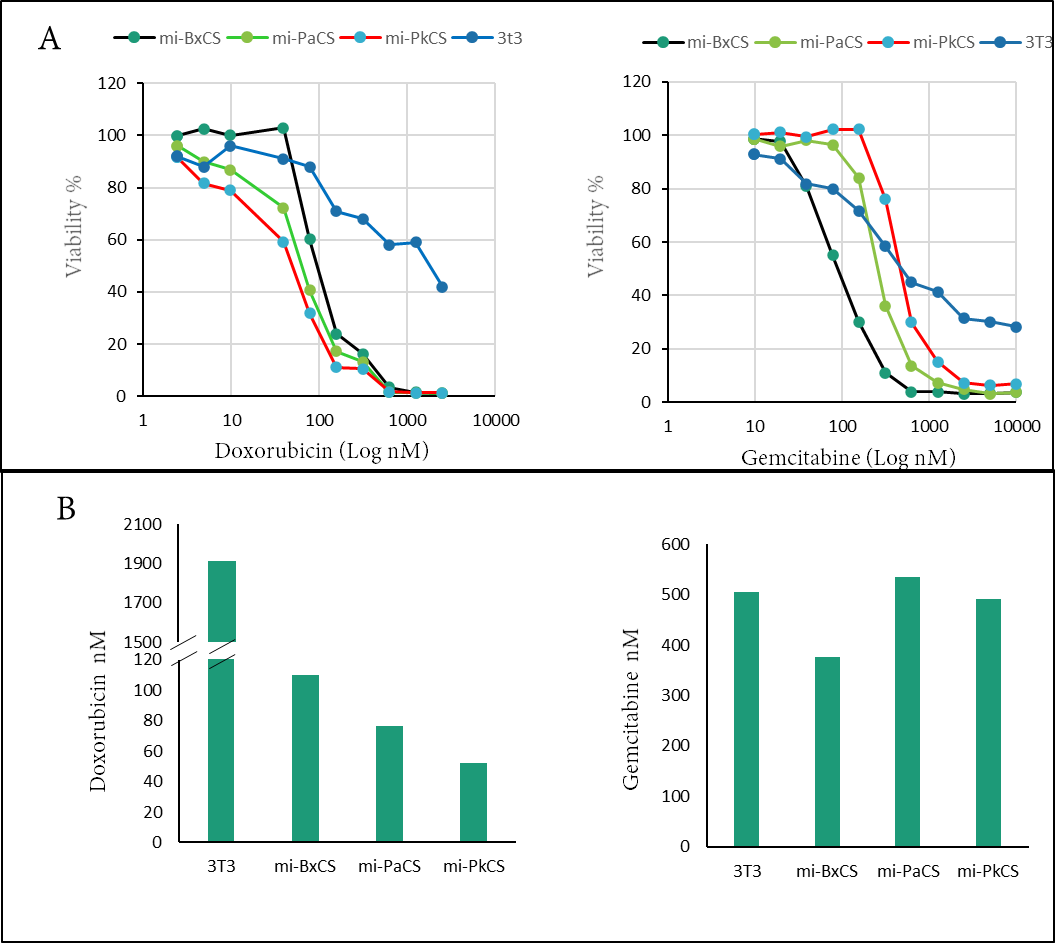


**Supplementary Figure 4.**

**A)** Dose–response curves of mi-BxCS, mi-PaCS, mi-PkCS and Balb/c 3T3 cells to doxorubicin and gemcitabine. Cells were treated with different concentrations of drugs for 72 hours and viability was quantified by MTT after the treatment. Each plot was taken from three independent experiments. Each plot is depicted as means ± SD. **B)** Bar graphs of the IC_50_s obtained from C) for doxorubicin and gemcitabine in mi-BxCS, mi-PaCS, mi-PkCS and Balb/c 3T3 cells.

**Supplementary Table 4. Gene expression count numbers of the most significant changing genes among pathways and metabolic related genes.**

| **Gene** | **miPSC** | **miBx** | **miPa** | **miPk** |
| --- | --- | --- | --- | --- |
| Actn3 | 135.08 | 216.30 | 704.11 | 835.78 |
| Ccnd2 | 3.99 | 73.98 | 205.30 | 210.59 |
| Col4a1 | 91.38 | 211.27 | 348.05 | 171.76 |
| Col4a2 | 37.76 | 169.42 | 204.50 | 175.05 |
| Ass1 | 88 | 190 | 412 | 278 |
| Atp5k | 156 | 289 | 694 | 777 |
| Atp6v0e2 | 55 | 107 | 221 | 146 |
| Azin2 | 149 | 414 | 761 | 700 |
| Galk1 | 1513 | 2069 | 5418 | 3574 |
| Gsk3a | 1054 | 850 | 990 | 884 |
| Lhpp | 38 | 104 | 196 | 195 |
| Ndufa2 | 367 | 711 | 1471 | 1289 |
| Ndufa3 | 232 | 425 | 639 | 571 |
| Ndufa4l2 | 196 | 770 | 967 | 746 |
| Ndufs3 | 66 | 104 | 210 | 182 |
| Ndufs6 | 313 | 770 | 970 | 798 |
| Ndufv3 | 743 | 996 | 1793 | 1378 |
| P4ha2 | 75 | 317 | 312 | 358 |
| Pcx | 354 | 565 | 1005 | 1335 |
| Pfkl | 3858 | 6211 | 12154 | 11088 |
| Pfkm | 153 | 690 | 411 | 392 |
| Pgam1 | 1105 | 2002 | 3177 | 2967 |
| Pgm2 | 1258 | 2192 | 4423 | 3459 |
| Tpi1 | 5727 | 8105 | 26489 | 17153 |
| Aldh1a1 | 3 | 2 | 0 | 0 |
| Aldh1a2 | 20 | 23 | 24 | 8 |
| Aldh1a3 | 7 | 4 | 1 | 0 |
| Aldh1a7 | 3 | 2 | 1 | 1 |
| Aldh16a1 | 424 | 364 | 744 | 494 |
| Aldh18a1 | 843 | 441 | 988 | 779 |
| Aldh1b1 | 97 | 92 | 144 | 51 |
| Aldh1l1 | 47 | 98 | 48 | 80 |
| Aldh1l2 | 50 | 67 | 25 | 13 |

**Supplementary Table 6. Gene expression count numbers of MDR genes**

| **Gene** | **miBx** | **miPa** | **miPk** |
| --- | --- | --- | --- |
| Abcb1b | 204 | 83 | 99 |
| Abcg2 | 564 | 390 | 305 |
| Abcc4 | 1561 | 224 | 182 |
| Abca1 | 134 | 22 | 31 |

**Supplementary Table 7**. **Antibody information**

| **Antibody** | **Company** | **Catalogue number** | **Dilution** | **Assay*** |
| --- | --- | --- | --- | --- |
| Anti-GFP (D5.1) rabbit mAb | Cell Signaling Technology, MA | 2956T | 1:200 | IHC |
| Anti-Ki67 Ab | Abcam, UK | ab833 | 1:50 | IHC |
| Anti-CD44 monoclonal antibody (IM7) | Thermo Fisher Scientific, MA. | 14-4031-82 | 1:200 | IHC |
| CD44 Antibody, anti-mouse, APC | Miltenyi Biotec, Germany | 130-102-563 | 1:10 | FC |
| CD24 Antibody, anti-mouse, APC | Miltenyi Biotec, Germany | 130-102-735 | 1:10 | FC |
| Isotype Control Antibody, rat IgG2b, APC | Miltenyi Biotec, Germany | 130-123-825 | 1:50 | FC |
| Anti-CD31 antibody | Abcam, UK | ab28364 | 1:200 | ICC |
| Goat anti-Rabbit IgG (H+L), Secondary Antibody, Alexa Fluor 555 | Thermo Fisher Scientific, MA. |  | 1:2000 | ICC |
| STEM121  Mouse Monoclonal Antibody Specific for Human Cytoplasmic Marker | Takara Bio, Japan | Y40410 | 1:1000 | IHC |
| Cytokeratin 7 antibody [N2C2], Internal | Genetex, CA | GTX110414 | 1:500 | IHC |
| CK20 Polyclonal Antibody | Bioss Antibodies, MA | bs-1588R | 1:400 | IHC |

* IHC: Immunohistochemistry, FC: flow cytometry, ICC: immunocytochemistry.

**Supplementary Table 8. Primer sets used in the study.**

| **Gene**  **Name** | **Accession Number** | **Forward Primer**  **5’ 🡪 3’** | **Reverse Primer**  **5’ 🡪 3’** |
| --- | --- | --- | --- |
| Alu repeats | **-** | GGTGAAACCCCGTCTCTACT | GGTTCAAGCGATTCTCCTGC* |
| Actb | NM_007393.5 | AAATCTGGCACCACACCTTC | GGGGTGTTGAAGGTCTCAAA |
| Nanog | NM_028016.3 | AGGGTCTGCTACTGAGATGCTCTG | CAACCACTGGTTTTTCTGCCACCG |
| Oct3/4 | NM_013633.3 | TCTTTCCACCAGGCCCCCGGCTC | TGCGGGCGGACATGGGGAGATCC |
| Sox2 | NM_011443.4 | TAGAGCTAGACTCCGGGCGATGA | TTGCCTTAAACAAGACCACGAAA |
| CD44 | NM_009851.2 | AGAAAAATGGCCGCTACAGTATC | TGCATGTTTCAAAACCCTTGC |
| Cxcr4 | NM_009911.3 | ACCAACAGTCAGAGGCCAAG | TGCTGGAATTGAAACACCAC |

*****Referred to Schneider, P.M.; Zhang, L.; Esdar, C.; Rittner, G.; Batzer, M.A.; Rittner, C. PCR Typing of Alu Elements — Molecular Genetics and Forensic Application. In Proceedings of 16th Congress of the International Society for Forensic Haemogenetics (Internationale Gesellschaft für forensische Hämogenetik e.V.), Santiago de Compostela, 12–16 September 1995, Berlin, Heidelberg, 1996//; pp. 118-120.
